# Distinct structure-function relationships across cortical regions and connectivity scales in the rat brain

**DOI:** 10.1038/s41598-019-56834-9

**Published:** 2020-01-09

**Authors:** Milou Straathof, Michel R. T. Sinke, Theresia J. M. Roelofs, Erwin L. A. Blezer, R. Angela Sarabdjitsingh, Annette van der Toorn, Oliver Schmitt, Willem M. Otte, Rick M. Dijkhuizen

**Affiliations:** 1 Biomedical MR Imaging and Spectroscopy Group, Center for Image Sciences, University Medical Center Utrecht and Utrecht University, Utrecht, The Netherlands; 2 Department of Translational Neuroscience, UMC Utrecht Brain Center, University Medical Center Utrecht and Utrecht University, Utrecht, the Netherlands; 3 Department of Anatomy, University of Rostock, Rostock, Germany; 4 Department of Pediatric Neurology, UMC Utrecht Brain Center, University Medical Center Utrecht and Utrecht University, Utrecht, the Netherlands

**Keywords:** Neural circuits, Brain

## Abstract

An improved understanding of the structure-function relationship in the brain is necessary to know to what degree structural connectivity underpins abnormal functional connectivity seen in disorders. We integrated high-field resting-state fMRI-based functional connectivity with high-resolution macro-scale diffusion-based and meso-scale neuronal tracer-based structural connectivity, to obtain an accurate depiction of the structure-function relationship in the rat brain. Our main goal was to identify to what extent structural and functional connectivity strengths are correlated, macro- and meso-scopically, across the cortex. Correlation analyses revealed a positive correspondence between functional and macro-scale diffusion-based structural connectivity, but no significant correlation between functional connectivity and meso-scale neuronal tracer-based structural connectivity. Zooming in on individual connections, we found strong functional connectivity in two well-known resting-state networks: the sensorimotor and default mode network. Strong functional connectivity within these networks coincided with strong short-range intrahemispheric structural connectivity, but with weak heterotopic interhemispheric and long-range intrahemispheric structural connectivity. Our study indicates the importance of combining measures of connectivity at distinct hierarchical levels to accurately determine connectivity across networks in the healthy and diseased brain. Although characteristics of the applied techniques may affect where structural and functional networks (dis)agree, distinct structure-function relationships across the brain could also have a biological basis.

## Introduction

The brain is a complex organ that can be regarded as a structural and functional network of interacting regions at the micro-, meso- and macroscopic level. At the macro-scale, whole-brain functional networks can be non-invasively mapped with resting-state functional MRI (resting-state fMRI). In resting-state fMRI data the inter-regional temporal correlations of spontaneous low-frequency blood oxygenation level-dependent (BOLD) fluctuations reflect functional connectivity^1,2^. Based on clusters of functionally connected regions, various resting-state networks have been identified, such as the default mode network^3^. These networks have been related to behavioral functioning in health and disease, and abnormalities partially explain pathophysiological processes and disease progression^4–6^.

The exact nature of functional connectivity is nonetheless not yet fully established. Since functional connectivity measured with resting-state fMRI relies on synchronous BOLD signals, understanding functional connectivity starts with understanding the origin of BOLD signals. The BOLD signal captures hemodynamic changes, such as blood flow, in response to neural activity. Although it is clear that BOLD signals reflect aspects of neural responses, it is still unclear which processes are the main contributors, i.e. excitation or inhibition, local field potentials, action potentials or multi-unit activity^7–9^. We know from primate and rodent studies that spontaneous BOLD fluctuations match with slow fluctuations in neuronal activity^10–12^. Moreover, in humans, BOLD signal fluctuations are related to slow cortical potentials and gamma band-limited power^13^. Still, the underlying structure of functional connectivity remains largely unknown. Since functional connectivity is found between adjacent and remote brain areas, short- and long-distance structural connections seem essential. Structural connectivity can be measured non-invasively with diffusion MRI and invasively with neuronal tracers. Diffusion-based tractography enables reconstruction of whole-brain macro-scale structural networks, by indirectly inferring the direction and strength of large white matter tracts from the diffusion of water^14,15^. In contrast, neuronal tracers use the transport mechanisms of cells to label existing mono- or polysynaptic connections. Tracers thus provide a direct and accurate measure of the directionality and strength at the meso-scale of individual axonal projections^16^.

Functional connectivity strength correlates with both diffusion- and neuronal tracer-based structural connectivity strength at the whole-brain level^17,18^; for an overview see^19^. However, different regions and connections display different structure-function relationships^20–22^. Identifying where and to what extent structural and functional connectivity strengths correlate will help to understand how brain networks are organized, and why functional abnormalities in brain disorders are related to characteristic patterns of disconnection or reorganization. So far, most studies have compared functional connectivity with structural connectivity measured at either the macro-scale or meso-scale, and thereby did not capture all aspects of structural connectivity. In addition, studies that applied diffusion MRI are hampered by the fact that a diffusion-based structural network is a suboptimal reconstruction of macro-scale axonal projections^23–26^. More accurate assessment of the structure-function relationships requires integration of functional connectivity with both macro-scale diffusion- and meso-scale neuronal tracer-based structural measures. Distinct structure-function relationships may be present at these different hierarchical levels^27^. Rodents are excellent species to study these relationships as resting-state fMRI and diffusion MRI-based tractography are feasible in rodents^28^ and comprehensive rodent databases of neuronal tracer-based structural connectivity are available as well^29,30^.

In this study we combined high-field resting-state fMRI-based functional connectivity measurements and diffusion- as well as neuronal tracer-based structural connectivity measurements from the rat brain to spatially map the structure-function relationship at the macro- and meso-scale. Our main goal was to identify to what extent structural and functional connectivity strength are correlated, macro- and meso-scopically, across the rat brain, which could explain differences in the functional significance of connections and their contribution to network dysfunction in brain disorders. We distinguished interhemispheric and intrahemispheric connections, as well as specific functional networks (sensorimotor or default mode network).

## Methods

### Ethics statement

All experiments were approved by the Committee for Animal Experiments of the University Medical Center Utrecht, The Netherlands, and were conducted in agreement with European regulations (Guideline 86/609/EEC) and Dutch laws (‘Wet op de Dierproeven’, 1996).

### Animals

#### *In vivo* resting-state functional connectivity

Resting-state functional connectivity was measured in twelve healthy adult male Wistar rats with a weight of 479 ± 44 g (mean ± standard deviation (SD)), which were group-housed and used for an earlier described study^31^. All animals had *ad libitum* access to food and water and were housed under the same environmental conditions (temperature 22–24° and 12 h light/dark cycle with lights on at 7:00 AM).

#### Post-mortem diffusion-based structural connectivity

Diffusion-based structural connectivity was measured in ten healthy adult male Wistar rats with an age of around twelve weeks. These animals were previously used in another study^32^ and group-housed under standard environmental conditions (12 h light/dark cycle with lights on at 7:00 AM). Animals were sacrificed by an intraperitoneal pentobarbital injection followed by transcardial perfusion-fixation with 4% paraformaldehyde in phosphate-buffered saline, as previously described^32^. We extracted the brains by removing all extracranial tissue, while leaving them inside the skull, and placed these in a proton-free oil (Fomblin®) prior to MR imaging to minimize susceptibility artefacts.

### MRI acquisition

All MRI experiments were conducted on a 9.4 T horizontal bore Varian MR system (Palo Alto, CA, USA), equipped with a 400 mT/m gradient coil (Agilent).

#### *In vivo* resting-state functional connectivity

Before MRI, the animals were anesthetized (with 4% of isoflurane in air for induction). Endotracheal intubation was performed to mechanically ventilate the rats with 1.5% of isoflurane in a mixture of air and O_2_ (4:1). End-tidal CO_2_ was continuously monitored with a capnograph (Microcap, Oridion Medical 1987 Ltd., Jerusalem, Israel). The animals were placed in an animal cradle and immobilized in a specially designed stereotactic holder. During MRI, a feed-back controlled heating pad ensured that the body temperature of the rats was maintained at 37.0 ± 1.0 °C. Blood oxygen saturation and heart rate were monitored with a pulse-oximeter from signals recorded with an infrared sensor attached to the hind paw of the animal.

We used a home-built 90 mm diameter Helmholtz volume coil for radiofrequency transmission, and an inductively coupled 25 mm diameter surface coil for signal detection. Prior to resting-state fMRI acquisition we acquired an anatomical image for registration purposes using 3D balanced steady-state free precession (bSSFP) imaging with four phase-cycling angles (0°, 90°, 180°, 270°). The acquisition parameters were as follows: repetition time (TR)/echo time (TE) = 5/2.5 ms; flip angle = 20°; field-of-view (FOV) = 40 × 32 × 24 mm^3^; acquisition matrix = 160 × 128 × 96; image resolution = 250 µm isotropic. Total acquisition time = 12.5 min. Resting-state fMRI images were acquired with T_2_^*^-weighted blood oxygenation level-dependent (BOLD) single shot 3D gradient-echo Echo Planar Imaging (EPI). The acquisition parameters were as follows: TR/TE = 26.1/15 ms; flip angle = 13°; FOV = 32.4 × 32.4 × 16.8 mm^3^, Acquisition matrix = 54 × 54 × 28; Spatial Resolution = 600 µm isotropic. The acquisition time was 730.8 ms per volume, with a total of 800 volumes, resulting in a scan time of 9 minutes and 45 seconds.

#### Post-mortem diffusion-based structural connectivity

For diffusion MRI we used a custom-made solenoid coil with an internal diameter of 26 mm. High spatial and angular resolution diffusion imaging (HARDI) was performed with an 8-shot 3D EPI sequence. The acquisition parameters were as follows: TR/TE = 500/32.4 ms, Δ/δ = 15/4 ms; b-value = 3842 s/mm^2^; FOV = 19.2 × 16.2 × 33 mm^3^; Acquisition matrix = 128 × 108 × 220; spatial resolution: 150 × 150 × 150 µm^3^. Diffusion-weighting was executed in 60 non-collinear directions on a half sphere and included five b_0_ non-diffusion-weighted images, with a total scan time of 8 hours.

### MRI processing

All MRI analyses were performed using FMRIB’s Software Library (FSL) v5.0, unless otherwise stated.

#### Regions of interest

To enable the selection of regions of interest, the mean resting-state fMRI image of each dataset was first linearly registered (*FLIRT*^33,34^) to the anatomical image of the same animal, followed by non-linear registration (*FNIRT*^35^) to a custom-built 3D model of the Paxinos and Watson rat brain atlas^36,37^. For diffusion MRI, the average of the non-diffusion-weighted images of each individual rat was non-linearly registered to this rat brain atlas. These registrations were used to transform 106 cortical bilateral regions into individual diffusion MRI and resting-state fMRI spaces. We only included regions of interest with sufficient assurance of spatial alignment, i.e. regions consisting of at least 8 voxels in individual resting-state fMRI space. This resulted in 82 bilateral cortical regions (Supplementary Table S1).

#### *In vivo* resting-state functional connectivity

The first twenty images of the resting-state fMRI scan were removed to ensure a steady state and the remaining images were motion-corrected to the mean volume with *MCFLIRT*^34^ and brain-extracted with *BET*^38^. The six motion correction parameters were used as regressors for the resting-state fMRI signal. No global signal regression was performed. Low-frequency BOLD fluctuations were obtained by band-pass filtering between 0.01 and 0.1 Hz in *AFNI*^39^. We performed an independent component analysis^40^ with 20 components to identify resting-state networks in the rat brain (Supplementary Fig. S1). To determine functional connectivity between brain regions, we calculated Fisher’s Z-transformed full correlation coefficients between the time-series for all pairs of regions of interest. These Fisher’s Z-transformed correlation coefficients were averaged over all rats to obtain a group-level measurement of functional connectivity strength between our regions of interest.

#### Post-mortem diffusion-based structural connectivity

We used single shell constrained spherical deconvolution (CSD) to construct a fiber orientation distribution (FOD) map for every rat. Next, CSD-based tractography, using the iFOD2 algorithm, was performed in MRtrix3® (http://www.mrtrix.org/)^41,42^. The iFOD2 algorithm uses 2^nd^ order integration over adjacent orientation distributions^42^. Whole brain tractography was done in individual subject space using dynamic seeding, thereby generating 2.5 million streamlines with a step size of 75 µm, an angle threshold of 40° and a FOD threshold of 0.2. The generated tractograms were filtered by *Spherical deconvolution Informed Filtering of Tracts* (SIFT)^43,44^. Subsequently, the connectomes were constructed by matching the whole-brain filtered tractograms with the regions of interest in subject space, by applying the registration procedure described above. Regions of interest were structurally connected if one or multiple streamlines had their endpoints in both regions, where the filtered number of inter-regional streamlines was indicative of structural connectivity strength. Finally, we calculated an average weighted connectome, in which the edge values represent structural connectivity strengths, to obtain a group-level measurement of diffusion-based structural connectivity strength between our regions of interest.

### Neuronal tracer-based structural connectivity

Neuronal tracer-based structural connectivity data was extracted from the NeuroVIISAS database^30^. This database contains rat nervous system data from over 7860 published tract-tracing studies, describing in total 591,435 ipsi- and contralateral connections. Many of these connections are described in multiple studies, affirming the robustness of the dataset. Studies with anterograde as well as retrograde monosynaptic tracers have been included, giving directionality information about the structural connections.

We used the same regions of interest as described for the functional connectivity and diffusion-based structural connectivity analyses to extract neuronal tracer-based structural connectivity for all pairs of regions (Supplementary Table S1). The weights of the directed connections are assigned in the NeuroVIISAS database as follows: 0: no connection or no information available; 1: light/sparse connection; 2: moderate/dense connection; 3: strong connection and 4: very strong connection.We averaged these connection weights over all studies investigating the same connection, resulting in a scale for neuronal tracer-based structural connectivity between 0 and 4.

### Experimental design and statistical analysis

**Figure 1:**
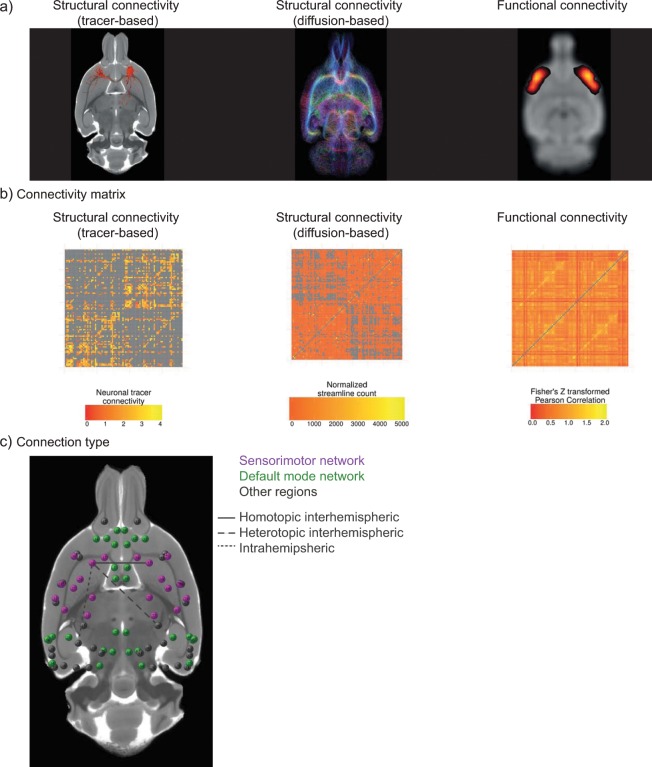
Overview of the analysis pipeline. Rat brain images are shown as axial views. Different measures of connectivity in the rat brain were assessed (**a**): meso-scale neuronal tracer-based structural connectivity (left), macro-scale diffusion-based structural connectivity (middle) and macro-scale resting-state functional connectivity (right). For each measure, we determined the connectivity matrix between 82 cortical regions of interest, with exclusion of the self-connections (central diagonal line) (**b**). We combined all connectivity matrices to determine the structure-function connectome of the rat brain (**c**) (circles representing nodes). The connectomes were reconstructed in 3D but are visualized in 2D. The colors in (**c**) represent two well-described functional resting-state networks in the rat brain: the sensorimotor network (purple) and the default mode network (green). Regions not belonging to these networks are shown in gray. The lines represent different regional types of connections: homotopic interhemispheric connection (solid line), heterotopic interhemispheric connection (dashed line) or intrahemispheric connection (dotted line) (see Methods section for an explanation of these connection types).

The analysis pipeline is illustrated in Fig. 1. All statistical and descriptive analyses were performed in R (version 3.2.3)^45^.

The network of 82 regions consisted of 6,724 directed connections, of which we removed the self-connections, resulting in a total of 6,642 unique connections. For the resting-state functional connectivity and diffusion-based structural connectivity networks, which did not contain directionality information, the network consisted of 3,321 unique connections. Only connections that existed in both the macro- and mesoscale structural connectivity datasets, meaning that they had a structural connectivity strength higher than zero in both datasets, were included for the analysis. In this way, we excluded connections with no information available in the neuronal tracer database, and minimized the amount of false-positives often present in diffusion-based tractography networks^24^.

#### Relationship between structural and functional connectivity strength at whole-brain level

To map the structure-function relationship globally, we performed correlation analyses between functional connectivity strength and macro-scale diffusion-based or meso-scale neuronal tracer-based structural connectivity separately. We applied a natural logarithmic transformation to both structural connectivity weights because they were skewed towards smaller connectivity weight values. Since the functional connectivity dataset and logarithmically transformed diffusion-based and neuronal tracer-based structural connectivity datasets were not normally distributed, we calculated a two-tailed Spearman rank correlation coefficient (ρ) between functional connectivity strength and logarithmically transformed macro-scale diffusion-based or meso-scale neuronal tracer-based structural connectivity strength. In addition, we calculated the correlation between functional and diffusion-based and neuronal tracer-based structural connectivity for interhemispheric and intrahemispheric connections separately. We determined the 95% confidence intervals of all Spearman rank correlation coefficients by means of bootstrapping with 5000 replicates. In addition, we determined whether functional connectivity strength was different between intrahemispheric and interhemispheric connections with a Wilcoxon rank sum test.

#### Relationship between structural and functional connectivity strength at connection level

To map the level of agreement between structural and functional connectivity at connection level, we selected the strongest and weakest structural connections at both the macro- and meso-scale. The strongest structural network was defined by connections that belonged to the 25% strongest diffusion-based and 25% strongest neuronal tracer-based structural connections. The weakest structural network was defined by connections that belonged to the 25% weakest diffusion-based and 25% weakest neuronal tracer-based structural connections. By combining macro- and meso-scale structural connectivity strengths, we selected the structural networks that were strong or weak at the level of individual axonal projections as well as at the level of large white matter bundles. This heightened the reliability of our assessment of the strength of structural connections and reduced the influence of methodological bias for specific connections. We compared functional connectivity strength between the strongest and weakest structural networks with a Wilcoxon rank sum test.

For both the strongest and weakest structural network, we determined the 25% strongest and 25% weakest functional connections, resulting in four sub-groups of connections. Two of these sub-groups represent connections where structural and functional connectivity strength agree: strong structural and functional connectivity or weak structural and functional connectivity. The other two subgroups are connections where structural and functional connectivity strength disagree: strong structural connectivity but weak functional connectivity or weak structural connectivity but strong functional connectivity.

To determine whether these subgroups of connections share common characteristics, we determined the Euclidian distance and type of connections and regions for all connections.

Between each pair of regions, we calculated the Euclidian distance, which is the shortest distance between two points in space (i.e. in a straight line). We determined the Euclidian distance for each pair of brain regions, because both structural and functional connectivity depend on distance, with in general lower connectivity for longer distances^46–48^. Therefore, we determined the x, y and z coordinate in mm of the center of gravity of each region in atlas space. Subsequently, we calculated the Euclidean distance, between each pair of regions i and j, with the following formula:

$$d(i,j)=\sqrt{{({x}_{i}-{x}_{j})}^{2}+{({y}_{i}-{y}_{j})}^{2}+{({z}_{i}-{z}_{j})}^{2}}$$

We divided the included connections and regions in sub-groups based on two different criteria. First, for each connection, we identified whether it was an intrahemispheric connection, which runs between two regions in the same hemisphere, or an interhemispheric connection, which runs between two regions in different hemispheres. In addition, we subdivided the interhemispheric connections into homotopic interhemispheric connections, which run between two homologous areas in different hemispheres, and heterotopic interhemispheric connections, which run between two dissimilar areas in different hemispheres (Fig. 1C). Second, for each region of interest, we assessed whether it belonged to one of two well-described functional networks in the rat brain, which were identified as the two networks explaining most of the variance in the independent component analysis of the functional connectivity dataset (Supplementary Fig. S1): the sensorimotor network or the default mode network (Fig. 1C). The sensorimotor network was defined as consisting of the left and right primary and secondary motor cortex (M1 and M2), subdivisions of the primary somatosensory cortex (S1BF, S1DZ, S1FL, S1HL, S1J, S1Tr, S1ULp) and the secondary somatosensory cortex (S2)^49^. The default mode network was defined as consisting of the left and right medial prefrontal cortex (mPFC), the cingulate cortex (Cg1 and Cg2), the orbital cortex (VO, MO and LO), the auditory/temporal association cortex (Au1, AuD, AuV and TeA), the posterior parietal cortex (ParPD) and the retrosplenial cortex (RSd, RSGb, RSGc)^50^. For each connection, we determined whether the connection was within one of these functional networks, or whether it was connecting one of these functional networks with another functional network.

## Results

Of all the possible 6,642 connections between the 82 selected regions of the cortical network, 1,175 connections (17.7% of the possible 6,642 connections) displayed structural connectivity in both the diffusion MRI and the neuronal tracer dataset. The average Euclidean distance for all the included connections in this network was 6.08 ± 3.35 mm (mean ± standard deviation (SD)).

### Global correlation between structural and functional connectivity depends on method and scale

**Figure 2:**
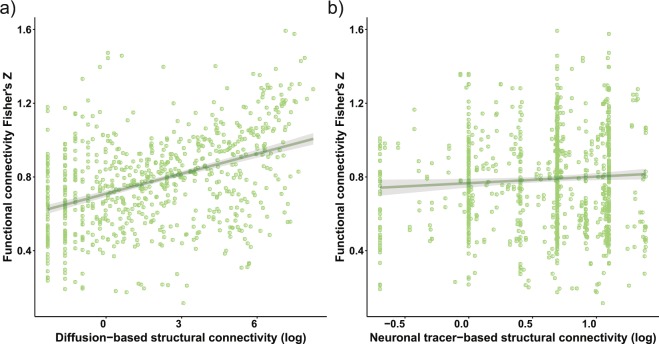
Whole-brain structure-function relationships at the structural macro-scale (diffusion-based structural connectivity) and meso-scale (neuronal tracer-based structural connectivity). Functional connectivity strength is plotted as the Fisher’s Z-transformed correlation coefficient versus the natural logarithmically transformed diffusion-based (**a**) or neuronal tracer-based structural connectivity strength (**b**). Individual connections are plotted as green circles. The structure-function relationship is shown as a linear fit, with shading representing the 95% confidence intervals of the fit.

Functional connectivity strength was positively correlated with diffusion-based structural connectivity strength in cortical connections (ρ = 0.41; p < 0.0001; 95% confidence interval: 0.36–0.46; Fig. 2a). For the same cortical connections, functional connectivity strength did not significantly correlate with neuronal tracer-based structural connectivity strength (ρ = 0.04; p = 0.14; 95% confidence interval: −0.01–0.10; Fig. 2b). In addition, we determined the structure-function correlation for interhemispheric and intrahemispheric connections separately. For interhemispheric connections, functional connectivity was significantly positively correlated with diffusion-based structural connectivity (ρ = 0.41; p < 0.0001; 95% confidence interval: 0.30–0.51) and with neuronal tracer-based structural connectivity (ρ = 0.25; p < 0.0001; 95% confidence interval: 0.14–0.35). In intrahemispheric connections, functional connectivity was significantly positively correlated with diffusion-based structural connectivity (ρ = 0.51; p < 0.0001; 95% confidence interval: 0.46–0.57), but not with neuronal tracer-based structural connectivity (ρ = −0.02; p = 0.64; 95% confidence interval: −0.08–0.05). In addition, functional connectivity was slightly higher in interhemispheric compared to intrahemispheric connections, although the distribution of functional connectivity values in these structural categories almost completely overlapped (interhemispheric connections: Fisher’s Z = 0.85 ± 0.28 (mean ± standard deviation); intrahemispheric connections: Fisher’s Z = 0.77 ± 0.25; p = 0.0002) (Supplementary Fig. S2).

### Different pathways and brain circuits display distinct structure-function relationships

**Figure 3:**
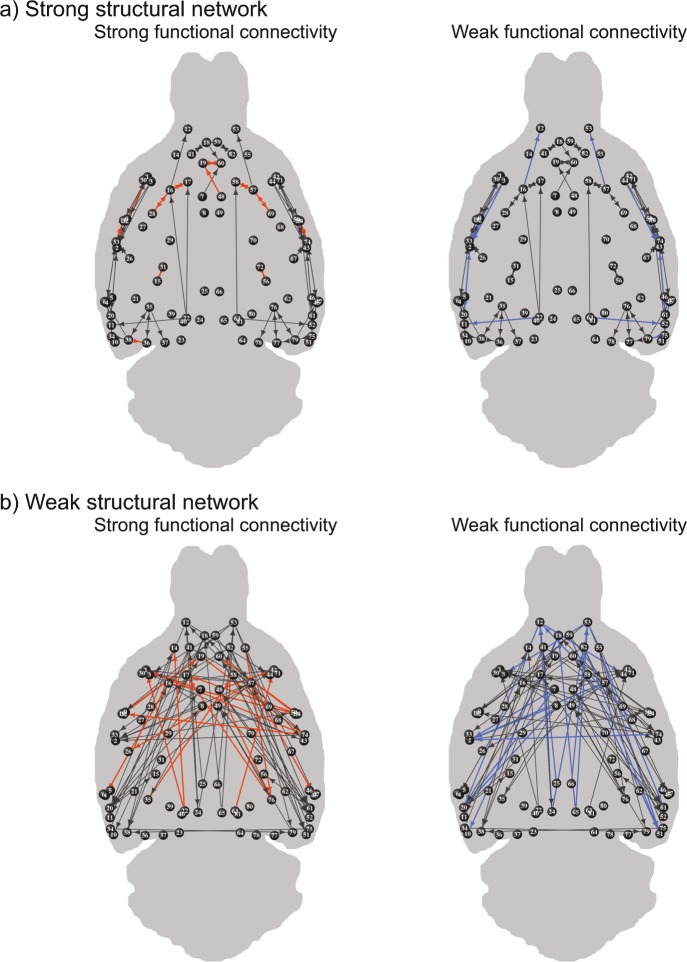
Strongest and weakest functional connections within the strongest and weakest structural networks. The strongest structural network consists of the connections that belong to both the 25% strongest structural connections at the macro-scale and the 25% strongest connections at the meso-scale (**a**), and the weakest structural network consists of the connections belonging to the 25% weakest at both hierarchical scales (**b**). Functional connectivity is orange colored for the 25% strongest (left) and blue colored for the 25% weakest functional connections (right). Circles represent the nodes (regions of interest), with numbers representing the regions listed in Supplementary Table S1, and lines represent the edges (connections). The connectomes were reconstructed in 3D but are visualized in 2D. The arrowheads reflect directionality information determined from the neuronal tracer-based structural connectivity dataset.

The strongest structural network at the macro- and meso-scale consisted of 107 cortical connections. These strongest structural connections were mainly intrahemispheric (93% of strongest structural network; left: 47%, right: 46%) with an average Euclidean distance of 2.54 ± 1.71 mm (see Fig. 3). The weakest structural network at the macro- and meso-scale consisted of 93 connections. Of these weakest structural connections, 31% was interhemispheric and 69% was intrahemispheric (left: 32%, right: 37%), with an average distance of 9.55 ± 2.42 mm (see Fig. 3). Functional connectivity was higher in the strongest structural network compared to the weakest structural network (Strong: Fisher’s Z = 0.94 ± 0.26; Weak: Fisher’s Z = 0.65 ± 0.21; p < 0.0001) (Supplementary Fig. S3).

**Figure 4:**
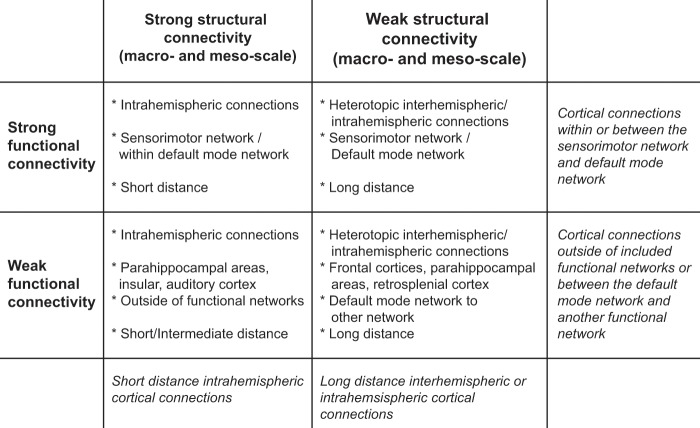
Characteristics of connections per subcategory of structural and functional connectivity. Structural connectivity is depicted in columns, whereas functional connectivity is depicted in rows. Strong connections belong to the 25% strongest connections; structurally based on diffusion MRI and neuronal tracing (i.e. at both the macro- and meso-scale) and functionally based on resting-state fMRI. Similarly, weak structural connections belong to the 25% weakest connections.

Within both the strongest and weakest cortical structural networks, we determined the 25% strongest and 25% weakest functional connections. These strongest and weakest functional connections are depicted in Fig. 3. The characteristics of these subcategories of connections are summarized in Fig. 4 and described below.

Connections with strong structural and functional connectivity are shown in Table 1. The average length of the connections was 1.34 ± 0.69 mm. Eighty-eight percent of these strongest connections was intrahemispheric (left: 50%; right: 38%). Sixty-two percent of the connections was part of the sensorimotor network. The homotopic connection between the left and right medial prefrontal cortex, which is part of the default mode network, was also one of the identified strongest connections.

**Table 1:** Characteristics of cortical connections in the rat brain with strong meso- and macro-scale structural connectivity and strong functional connectivity.

Seed	Target	Neuronal tracer-based structural connectivity strength	Diffusion-based structural connectivity strength	Functional connectivity strength (Z’)	Euclidean distance (mm)	Connection type (network)	Connection type (regional)
Left mPFC	Right mPFC	3.00	1790.10	1.58	1.18	Within default mode network	Homotopic interhemispheric
Right mPFC	Left mPFC	3.00	1790.10	1.58	1.18	Within default mode network	Homotopic interhemispheric
Left DI	Left GI	2.96	1046.70	1.30	0.31	No	Intrahemispheric left
Left GI	Left DI	2.89	1046.70	1.30	0.31	No	Intrahemispheric left
Left LPtA	Left S1Tr	3.00	629.20	1.28	0.97	Sensorimotor network to another network	Intrahemispheric left
Left S1Tr	Left LPtA	3.00	629.20	1.28	0.97	Sensorimotor network to another network	Intrahemispheric left
Left V2L	Left V1B	3.00	1474.60	1.28	1.43	No	Intrahemispheric left
Left M1	Left M2	2.96	3787.60	1.28	1.27	Within sensorimotor network	Intrahemispheric left
Left M2	Left M1	3.76	3787.60	1.28	1.27	Within sensorimotor network	Intrahemispheric left
Right LPtA	Right S1Tr	3.00	533.70	1.27	0.98	Sensorimotor network to another network	Intrahemispheric right
Right S1Tr	Right LPtA	3.00	533.70	1.27	0.98	Sensorimotor network to another network	Intrahemispheric right
Left GI	Left S2	3.72	1075.40	1.25	1.64	Sensorimotor network to another network	Intrahemispheric left
Left S2	Left GI	3.62	1075.40	1.25	1.64	Sensorimotor network to another network	Intrahemispheric left
Right M1	Right M2	2.96	2958.90	1.22	1.27	Within sensorimotor network	Intrahemispheric right
Right M2	Right M1	3.76	2958.90	1.22	1.27	Within sensorimotor network	Intrahemispheric right
Right GI	Right S2	3.72	1261.10	1.22	1.63	Sensorimotor network to another network	Intrahemispheric right
Right S2	Right GI	3.62	1261.10	1.22	1.63	Sensorimotor network to another network	Intrahemispheric right
Right DI	Right GI	2.96	1200.00	1.21	0.31	No	Intrahemispheric right
Right GI	Right DI	2.89	1200.00	1.21	0.31	No	Intrahemispheric right
Left M1	Left S1FL	2.88	1189.60	1.20	1.90	Within sensorimotor network	Intrahemispheric left
Left S1FL	Left M1	2.86	1189.60	1.20	1.90	Within sensorimotor network	Intrahemispheric left
Right M1	Right S1FL	2.88	1077.70	1.20	1.91	Within sensorimotor network	Intrahemispheric right
Right S1FL	Right M1	2.86	1077.70	1.20	1.91	Within sensorimotor network	Intrahemispheric right
Left AuD	Left Au1	3.00	1098.90	1.18	0.78	Within default mode network	Intrahemispheric left
Right Cg1	Left mPFC	3.00	260.00	1.18	2.87	Within default mode network	Heterotopic interhemispheric
Left DI	Left AID	3.00	851.00	1.16	3.05	No	Intrahemispheric left

Seed and target regions were determined from the NeuroVIISAS tracer database. AID: agranular insular cortex dorsal part; Au1: primary auditory cortex; AuD: secondary auditory cortex dorsal area; Cg1: cingulate cortex area 1; DI: dysgranular insular cortex; GI: granular insular cortex; LPtA: lateral parietal association cortex; M1: primary motor cortex; M2: secondary motor cortex; mPFC: medial prefrontal cortex; S1FL: primary somatosensory cortex forelimb region; S1Tr: primary somatosensory cortex trunk region; S2: secondary somatosensory cortex; V1B: primary visual cortex binocular area; V2L: secondary visual cortex lateral area.

Table 2 shows the connections that we identified as belonging to the 25% weakest structural and functional connections. The average length of the weakest structural and functional connections was 10.84 ± 2.09 mm. The identified connections included 30% heterotopic interhemispheric and 70% intrahemispheric (left: 44%, right: 26%) connections, and were mainly between frontal cortices, parahippocampal areas and the retrosplenial cortex. Sixty-one percent of the connections connected the default mode network with another functional network.

**Table 2:** Characteristics of cortical connections in the rat brain with weak meso- and macro-scale structural connectivity and weak functional connectivity.

Seed	Target	Neuronal tracer-based structural connectivity strength	Diffusion-based structural connectivity strength	Functional connectivity strength (Z’)	Euclidean distance (mm)	Connection type (network)	Connection type (regional)
Right LO	Right DLEnt	0.50	0.20	0.19	12.49	Default mode network to another network	Intrahemispheric right
Right VO	Right DLEnt	0.90	0.30	0.23	12.85	Default mode network to another network	Intrahemispheric right
Left MO	Left DLEnt	1.00	0.20	0.25	13.99	Default mode network to another network	Intrahemispheric left
Right MO	Right DLEnt	1.00	0.10	0.26	14.00	Default mode network to another network	Intrahemispheric right
Left FrA	Left PRh	1.05	0.20	0.28	13.11	No	Intrahemispheric left
Right RSGb	Right FrA	0.50	0.20	0.33	11.97	Default mode network to another network	Intrahemispheric right
Right FrA	Right PRh	1.05	0.20	0.37	13.10	No	Intrahemispheric right
Left RSGb	Left FrA	0.50	0.50	0.39	11.95	Default mode network to another network	Intrahemispheric left
Right RSGc	Right FrA	0.50	0.40	0.40	10.26	Default mode network to another network	Intrahemispheric right
Left Cg2	Left AIP	0.50	0.30	0.41	7.61	Default mode network to another network	Intrahemispheric left
Left Cg1	Left AIP	0.50	0.10	0.43	8.50	Default mode network to another network	Intrahemispheric left
Left Cg2	Left PRh	1.00	0.20	0.44	9.54	Default mode network to another network	Intrahemispheric left
Left PRh	Left Cg2	1.00	0.20	0.44	9.54	Default mode network to another network	Intrahemispheric left
Right S1DZ	Left FrA	1.00	0.10	0.45	8.66	Sensorimotor network to another network	Heterotopic interhemispheric
Right FrA	Left AIV	1.13	0.30	0.45	7.12	No	Heterotopic interhemispheric
Left AIP	Right M1	0.74	0.20	0.45	11.15	Sensorimotor network to another network	Heterotopic interhemispheric
Left RSGc	Left FrA	0.50	0.40	0.46	10.24	Default mode network to another network	Intrahemispheric left
Left FrA	Right AIV	1.13	0.10	0.46	7.11	No	Heterotopic interhemispheric
Left Cg1	Left PRh	1.00	0.20	0.46	10.58	Default mode network to another network	Intrahemispheric left
Left PRh	Left Cg1	0.75	0.20	0.46	10.58	Default mode network to another network	Intrahemispheric left
Right S2	Left AIP	0.75	0.10	0.47	12.64	Sensorimotor network to another network	Heterotopic interhemispheric
Right RSGb	Left MO	0.50	0.10	0.47	11.36	Within default mode network	Heterotopic interhemispheric
Left RSGb	Right VO	0.50	0.10	0.47	10.90	Within default mode network	Heterotopic interhemispheric

Seed and target regions were determined from the NeuroVIISAS tracer database. AIP: agranular insular cortex posterior part; AIV: agranular insular cortex ventral part; Cg1: cingulate cortex area 1; Cg2: cingulate cortex area 2; DLEnt: dorsolateral entorhinal cortex; FrA: frontal association cortex; LO: lateral orbital cortex; M1: Primary motor cortex; MO: medial orbital cortex; Prh: perirhinal cortex; RSGb: retrosplenial granular cortex b region; RSGc: retrosplenial granular cortex c region; S1DZ: primary somatosensory cortex dysgranular region; S2: Secondary somatosensory cortex; VO: ventral orbital cortex.

Table 3 shows connections with strong structural but weak functional connectivity. All these connections were intrahemispheric (50% right; 50% left), of which the average Euclidean distance between regions was 3.23 ± 1.64 mm. Many of these connections were between parahippocampal areas and the insular cortex or auditory cortex, and within the insular cortex.

**Table 3:** Characteristics of cortical connections in the rat brain with strong meso- and macro-scale structural connectivity and weak functional connectivity.

Seed	Target	Neuronal tracer-based structural connectivity strength	Diffusion-based structural connectivity strength	Functional connectivity strength (Z’)	Euclidean distance (mm)	Connection type (network)	Connection type (regional)
Left AIP	Left PRh	3.66	317.50	0.42	4.37	No	Intrahemispheric left
Left PRh	Left AIP	4.00	317.50	0.42	4.37	No	Intrahemispheric left
Right TeA	Right PRh	2.94	250.70	0.46	2.38	Default mode network to another network	Intrahemispheric right
Left AuV	Left PRh	2.75	141.30	0.48	1.49	Default mode network to another network	Intrahemispheric left
Left TeA	Left PRh	2.94	466.60	0.51	2.38	Default mode network to another network	Intrahemispheric left
Right M1	Right FrA	2.93	154.80	0.52	4.17	Sensorimotor network to another network	Intrahemispheric right
Right AIP	Right PRh	3.66	316.30	0.53	4.35	No	Intrahemispheric right
Right PRh	Right AIP	4.00	316.30	0.53	4.35	No	Intrahemispheric right
Left Ect	Left PRh	3.90	1523.80	0.56	1.14	No	Intrahemispheric left
Left PRh	Left Ect	3.83	1523.80	0.56	1.14	No	Intrahemispheric left
Right S2	Right AIP	3.00	125.00	0.56	2.22	Sensorimotor network to another network	Intrahemispheric right
Left AIV	Left AIP	3.00	301.10	0.59	4.86	No	Intrahemispheric left
Left M1	Left FrA	2.93	181.70	0.59	4.18	Sensorimotor network to another network	Intrahemispheric left
Right Ect	Right PRh	3.90	1215.60	0.61	1.12	No	Intrahemispheric right
Right PRh	Right Ect	3.83	1215.60	0.61	1.12	No	Intrahemispheric right
Right RSd	Right Ect	3.00	226.80	0.62	6.12	Default mode network to another network	Intrahemispheric right
Left AID	Left AIP	3.00	145.90	0.63	5.20	No	Intrahemispheric left
Left AIP	Left AID	2.90	145.90	0.63	5.20	No	Intrahemispheric left
Right AIV	Right AIP	3.00	277.30	0.66	4.88	No	Intrahemispheric right
Right Ect	Right Au1	3.00	217.30	0.67	1.81	Default mode network to another network	Intrahemispheric right
Left AIP	Left GI	3.69	249.30	0.68	2.21	No	Intrahemispheric left
Left GI	Left AIP	2.91	249.30	0.68	2.21	No	Intrahemispheric left
Left RSd	Left Ect	3.00	152.40	0.71	6.12	Default mode network to another network	Intrahemispheric left
Right TeA	Right V2L	2.86	1078.80	0.71	2.17	Default mode network to another network	Intrahemispheric right
Right AIP	Right GI	3.69	354.00	0.72	2.20	No	Intrahemispheric right
Right GI	Right AIP	2.91	354.00	0.72	2.20	No	Intrahemispheric right

Seed and target regions were determined from the NeuroVIISAS tracer database. AID: agranular insular cortex dorsal part; AIP: agranular insular cortex posterior part; AIV: agranular insular cortex ventral part; Au1: Primary auditory cortex; AuV: Secondary auditory cortex ventral area; Ect: ectorhinal cortex; FrA: frontal association cortex; GI: granular insular cortex; M1: primary motor cortex; PRh: perirhinal cortex; RSd: Retrosplenial dorsal; S2: secondary somatosensory cortex; TeA: temporal association cortex 1; V1B: primary visual cortex binocular area.

The connections belonging to 25% strongest functional but 25% weakest structural connections are shown in Table 4. The average Euclidean length of the connections was 8.67 ± 1.78 mm and 35% were heterotopic interhemispheric, all of which were part of the sensorimotor network. Fifty-two percent of the connections resided within the sensorimotor network or between the sensorimotor network and another network, of which 42% connected the sensorimotor with the default mode network. Forty-three percent of the connections was between the default mode network and another functional network.

**Table 4:** Characteristics of cortical connections in the rat brain with weak meso- and macro-scale structural connectivity and strong functional connectivity.

Seed	Target	Neuronal tracer-based structural connectivity strength	Diffusion-based structural connectivity strength	Functional connectivity strength (Z’)	Euclidean distance (mm)	Connection type (network)	Connection type (regional)
Right S2	Left GI	1.00	0.30	1.14	12.12	Sensorimotor network to another network	Heterotopic interhemispheric
Right DI	Right mPFC	1.44	0.30	1.01	6.70	Default mode network to another network	Intrahemispheric right
Right M1	Right Au1	1.00	0.60	1.00	8.57	Default mode network to sensorimotor network	Intrahemispheric right
Right Au1	Right M1	1.00	0.60	1.00	8.57	Default mode network to sensorimotor network	Intrahemispheric right
Left M1	Left Au1	1.00	0.40	0.99	8.57	Default mode network to sensorimotor network	Intrahemispheric left
Left Au1	Left M1	1.00	0.40	0.99	8.57	Default mode network to sensorimotor network	Intrahemispheric left
Left DI	Left mPFC	1.44	0.20	0.98	6.71	Default mode network to another network	Intrahemispheric left
Right M1	Left S1BF	1.00	0.50	0.96	9.20	Within sensorimotor network	Heterotopic interhemispheric
Left GI	Left mPFC	1.43	0.20	0.95	6.84	Default mode network to another network	Intrahemispheric left
Right GI	Right mPFC	1.43	0.10	0.93	6.83	Default mode network to another network	Intrahemispheric right
Left V1	Right M2	1.00	0.20	0.92	9.63	Sensorimotor network to another network	Heterotopic interhemispheric
Left RSd	Left AID	1.00	0.10	0.90	9.90	Default mode network to another network	Intrahemispheric left
Right V1	Left M2	1.00	0.20	0.90	9.64	Sensorimotor network to another network	Heterotopic interhemispheric
Right Cg1	Right AID	0.50	0.10	0.89	5.28	Default mode network to another network	Intrahemispheric right
Left RSd	Left LO	0.50	0.10	0.89	10.65	Within default mode network	Intrahemispheric left
Right Cg1	Right V1	1.00	0.50	0.88	7.72	Default mode network to another network	Intrahemispheric right
Right S2	Left AID	0.75	0.10	0.87	11.21	Sensorimotor network to another network	Heterotopic interhemispheric
Right LO	Right V1	1.00	0.20	0.86	10.26	Default mode network to another network	Intrahemispheric right
Left M1	Right GI	1.07	0.50	0.85	9.70	Sensorimotor network to another network	Heterotopic interhemispheric
Right GI	Left M2	1.00	0.30	0.84	8.91	Sensorimotor network to another network	Heterotopic interhemispheric
Left Cg1	Left AID	0.50	0.10	0.83	5.28	Default mode network to another network	Intrahemispheric left
Left mPFC	Right S2	0.50	0.20	0.82	8.60	Default mode network to sensorimotor network	Heterotopic interhemispheric
Right RSd	Right AID	1.00	0.30	0.81	9.92	Default mode network to another network	Intrahemispheric right

Seed and target regions were determined from the NeuroVIISAS tracer database. AID: agranular insular cortex dorsal part; Au1: primary auditory cortex; Cg1: cingulate cortex area 1; DI: dysgranular insular cortex; GI: granular insular cortex; LO: lateral orbital cortex; M1: primary motor cortex; M2: secondary motor cortex; mPFC: medial prefrontal cortex; RSd: retrosplenial dorsal; S1BF: primary somatosensory cortex barrel field; S2: secondary somatosensory cortex; V1: primary visual cortex.

## Discussion

Our study on the rat brain shows that cortical brain networks are characterized by functional connectivity strengths, as measured with resting-state fMRI, that partly associate with macro-scale diffusion-based structural connectivity strength but not significantly associate with meso-scale neuronal tracer-based structural connectivity strength. When examining brain areas where structural and functional connectivity agreed or disagreed, we found that strong functional connectivity in the sensorimotor and default mode network matched with strong structural connectivity of intrahemispheric connections but was accompanied by weak structural connectivity of interhemispheric and long-range intrahemispheric connections.

### Distinct global structure-function relationships across different hierarchical levels of structural connectivity

The partial positive correspondence between functional connectivity and diffusion-based structural connectivity strength in the rat brain is in line with structure-function relationships found in humans^19^. However, we did not find a significant correlation between functional connectivity and meso-scale neuronal tracer-based structural connectivity strength. One previous study investigated this relationship at the meso-scale in rats and reported a positive structure-function correlation (r = 0.48)^51^. However, this study did not include essential interhemispheric connections. Interhemispheric connections are known to have lower structure-function relationships^52^, which may be explained by long inter-regional distances, sparser interhemispheric connectivity or involvement of polysynaptic or indirect connections^53^. Distinct structure-function relationships at the structural macro- and meso-scale have already been demonstrated in a study combining datasets in humans (functional and diffusion-based structural connectivity) and macaques (neuronal tracer-based structural connectivity)^27^. However, the authors could not disentangle whether these distinct relationships were due to species differences or due to different measures of structural connectivity. Since we compared all three measures in the same species, (dis)agreement between structural and functional connectivity most likely reflects topological differences in the structure-function relationship across different hierarchical levels.

Beside being measurements at different hierarchical levels, another important difference between macro-scale diffusion-based and meso-scale neuronal tracer-based structural connectivity is the directionality information available in the data. Whereas diffusion-based structural connectivity does not provide directionality information, meaning that all connections are considered to be fully reciprocal, neuronal tracer-based structural connectivity does provide this directionality information. Since resting-state functional connectivity is also directionless, the correlation of functional connectivity with diffusion-based structural connectivity may be higher than with neuronal tracer-based structural connectivity. In addition, the correlation between functional connectivity and diffusion-based structural connectivity may also be explained by the fact that both connectivity measures are determined with the same measurement tool, i.e. MRI.

### Strong functional connectivity in robust resting-state networks is supported by strong short-range intrahemispheric connections

The sensorimotor and default mode network are robustly established resting-state networks in the rodent brain^49,54^, which was corroborated by our finding of strong functional connectivity in or between these networks. We also observed strong short-range intrahemispheric structural connections at meso- and macro-scale in these networks. Strong reciprocal structural connections have previously been shown between ipsilateral sensorimotor cortices, measured with neuronal tracers^55–57^, and between default mode network regions, measured with diffusion MRI^58,59^. In comparison, in the current study we found that heterotopic interhemispheric structural connections in the sensorimotor network and long-range intrahemispheric structural connections between the default mode network and other functional networks were weak at both the macro- and the meso-scale. Since both connection types were between areas located far apart from each other, this observation may reflect the difficulties of diffusion-based tractography to reconstruct long-distance connections^46^, and the distance-dependence of neuronal tracer-based structural connectivity strength^47^. Hereby, our data point out that the distance-dependence of structural connectivity strength, as determined from diffusion MRI or neuronal tracing, influences measurements of structure-function relationships. This should be taken into account in studies on the relation between structural and functional connectivity. However, weak heterotopic interhemispheric connectivity may also reflect the smaller role these connections play in functional brain organization as compared to homotopic interhemispheric connections^60,61^. Interestingly, strong functional connectivity in homotopic interhemispheric connections within the sensorimotor network was not accompanied by strong structural connectivity, despite the presence of a large bundle of neuronal fibers, i.e. the corpus callosum, connecting the two hemispheres. This may be a result of our approach of only including connections that exhibit macro- and meso-scale structural connectivity. Homotopic interhemispheric connections in the sensorimotor network were included in the 25% strongest meso-scale neuronal tracer-based structural network, but not in the 25% strongest macro-scale diffusion-based structural network. Therefore, we limit our conclusions to connections with matching macro- and meso-scale structural connectivity, while other structure-function relationships may exist in connections where macro- and meso-scale structural connectivity do not match.

### Implications of different structure-function relationships across the brain in health and disease

We have shown that distinct structure-function relationships exist in different cortical connections of the rat brain, in line with a previous study reporting that 25% of valid structural connections are very weak functional connections^62^. Different structure-function relationships can have implications for brain functioning and behavior. Healthy brain functioning relies on a balance between segregation and integration of neuronal communications^63,64^. Structure-function relationships have been shown to be stronger when functional networks are in an integrated state, compared to a segregated state^65^. In another study, white matter integrity was associated with BOLD signal complexity in local connections (structure-function agreement) but not in distributed connections (structure-function disagreement)^66^. This suggests that information integration relies on a strong structure-function relationship, whereas weak structure-function relationships are implied in segregation.

Next to the implication of structure-function relationships on healthy brain functioning, structure-function relationships may (partly) determine the functional effects of structural damage to the brain. Intuitively, it may be deduced that structural damage to connections with strong structure-function relationships will have severer functional consequences than structural damage to connections with weak structure-function relationships. Novel algorithms may enable us to predict the functional effects of specific structural damage^67^. Alterations and preservations of structural and functional connectivity in human patients, and in animal models of neurological and psychiatric diseases, can provide insights into the impact of structure-function couplings on outcome. For example, after stroke significant changes in structural and functional connectivity have been measured in the remaining intact sensorimotor network in rodents^68–70^ and humans^71–73^. Chronically after experimental stroke in rats, structural and functional connectivity changes were related intrahemispherically –on the side of the stroke lesion– while this was not evident for interhemispheric connections^68^. This may be explained by a stronger structure-function agreement in intrahemispheric sensorimotor connections as compared to interhemispheric sensorimotor connections, as we found in the current study.

A strength of the current study is the inclusion of three different measures of connectivity within a single species. Comparing functional connectivity against macro-scale diffusion-based as well as meso-scale neuronal tracer-based structural connectivity in rats enabled the investigation of structure-function relationships across hierarchical levels. In addition, by including both diffusion- and neuronal tracer-based structural connectivity measures, we could avoid inclusion of false positives that are often present in diffusion-based structural networks^24,25,74,75^. A reliable structural network of the rat brain was created by only selecting connections present in both diffusion- and neuronal tracer-based structural networks. The relationship between diffusion-based structural connectivity and resting-state functional connectivity may have been higher when both measures would have been acquired in the same rat. However, neuronal tracer-based structural connectivity was acquired from many different groups of rats. Therefore, we also measured diffusion-based structural connectivity in a separate group of rats, to prevent inappropriate comparison with potentially higher within-subject correlations. A limitation could be the restriction of our assessments to monosynaptic connections. In addition, resting-state functional connectivity was determined under anesthesia, which influences functional connectivity measures^76^ and possibly affects the structure-function relationship.

In conclusion, we demonstrated a correlation between functional connectivity and diffusion-based structural connectivity, but no significant correlation between functional connectivity and neuronal tracer-based structural connectivity in the rat cortex. These distinct structure-function relationships may be due to different hierarchical levels of measurement or directionality information available in the data. In addition, the structure-function relationship varies across cortical regions in the rat brain. Characteristics of the used techniques, such as distance-dependency, affect where structural and functional networks (dis)agree. Conclusions about connectivity based on a single technique may therefore be biased. This shows the importance of combining different complementary measures of connectivity at distinct hierarchical levels to accurately determine connectivity across networks in the healthy and diseased brain.

## Supplementary information

Supplementary Materials.

## Data Availability

The datasets generated during and/or analyzed during the current study are available from the corresponding author on reasonable request.
