# Supplementary material for: Distinct structure-function relationships across cortical regions and connectivity scales in the rat brain
Source: Sci Rep. 2020 Jan 9;10:56. doi: 10.1038/s41598-019-56834-9 (PMC6952407; doi:10.1038/s41598-019-56834-9)
Supplement: Supplementary file 1 — Supplementary Materials. [file 41598_2019_56834_MOESM1_ESM.docx]

**Distinct structure-function relationships across cortical regions and connectivity scales in the rat brain**

Milou Straathof ^1 *^, Michel R.T. Sinke ^1^, Theresia J.M. Roelofs ^1,2^, Erwin L.A. Blezer ^1^, R. Angela Sarabdjitsingh ^2^, Annette van der Toorn ^1^, Oliver Schmitt ^3^, Willem M. Otte ^1,4^ & Rick M. Dijkhuizen ^1 *^

**Supplementary Table S1: Included regions of interest for resting-state fMRI, diffusion MRI and neuronal tracer analyses.**

| *Names (Abbreviations) of Paxinos & Watson atlas regions* | *Left* | *Right* |
| --- | --- | --- |
| *Agranular insular cortex dorsal part (AID)* | 1 | 42 |
| *Agranular insular cortex posterior part (AIP)* | 2 | 43 |
| *Agranular insular cortex ventral part (AIV)* | 3 | 44 |
| *Primary auditory cortex (Au1)* | 4 | 45 |
| *Secondary auditory cortex dorsal area (AuD)* | 5 | 46 |
| *Secondary auditory cortex ventral area (AuV)* | 6 | 47 |
| *Cingulate cortex area 1 (Cg1)* | 7 | 48 |
| *Cingulate cortex area 2 (Cg2)* | 8 | 49 |
| *Dysgranular insular cortex (DI)* | 9 | 50 |
| *Dorsolateral entorhinal cortex (DLEnt)* | 10 | 51 |
| *Ectorhinal cortex (Ect)* | 11 | 52 |
| *Frontal association cortex (FrA)* | 12 | 53 |
| *Granular insular cortex (GI)* | 13 | 54 |
| *Lateral orbital cortex (LO)* | 14 | 55 |
| *Lateral parietal association cortex (LptA)* | 15 | 56 |
| *Primary motor cortex (M1)* | 16 | 57 |
| *Secondary motor cortex (M2)* | 17 | 58 |
| *Medial orbital cortex (MO)* | 18 | 59 |
| *Medial prefrontal cortex (mPFC)* | 19 | 60 |
| *Perirhinal cortex (Prh)* | 20 | 61 |
| *Parietal cortex posterior area dorsal part (ParPD)* | 21 | 62 |
| *Retrospenial dorsal (RSd)* | 22 | 63 |
| *Retrosplenial granular cortex a region (RSGa)* | 23 | 64 |
| *Retrosplenial granular cortex b region (RSGb)* | 24 | 65 |
| *Retrosplenial granular cortex c region (RSGc)* | 25 | 66 |
| *Primary somatosensory cortex barrel field (S1BF)* | 26 | 67 |
| *Primary somatosensory cortex dysgranular region (S1DZ)* | 27 | 68 |
| *Primary somatosensory cortex forelimb region (S1FL)* | 28 | 69 |
| *Primary somatosensory cortex hindlimb region (S1HL)* | 29 | 70 |
| *Primary somatosensory cortex jaw region (S1J)* | 30 | 71 |
| *Primary somatosensory cortex trunk region (S1Tr)* | 31 | 72 |
| *Primary somatosensory cortex upper lib region (S1ULp)* | 32 | 73 |
| *Secondary somatosensory cortex (S2)* | 33 | 74 |
| *Temporal association cortex 1 (TeA)* | 34 | 75 |
| *Primary visual cortex (V1)* | 35 | 76 |
| *Primary visual cortex binocular area (V1B)* | 36 | 77 |
| *Primary visual cortex monocular area (V1M)* | 37 | 78 |
| *Secondary visual cortex lateral area (V2L)* | 38 | 79 |
| *Secondary visual cortex mediolateral area (V2ML)* | 39 | 80 |
| *Secondary visual cortex mediomedial area (V2MM)* | 40 | 81 |
| *Ventral orbital cortex (VO)* | 41 | 82 |

The numbers for the left and right regions of interest are corresponding to the numbers of the nodes in Figure 2.


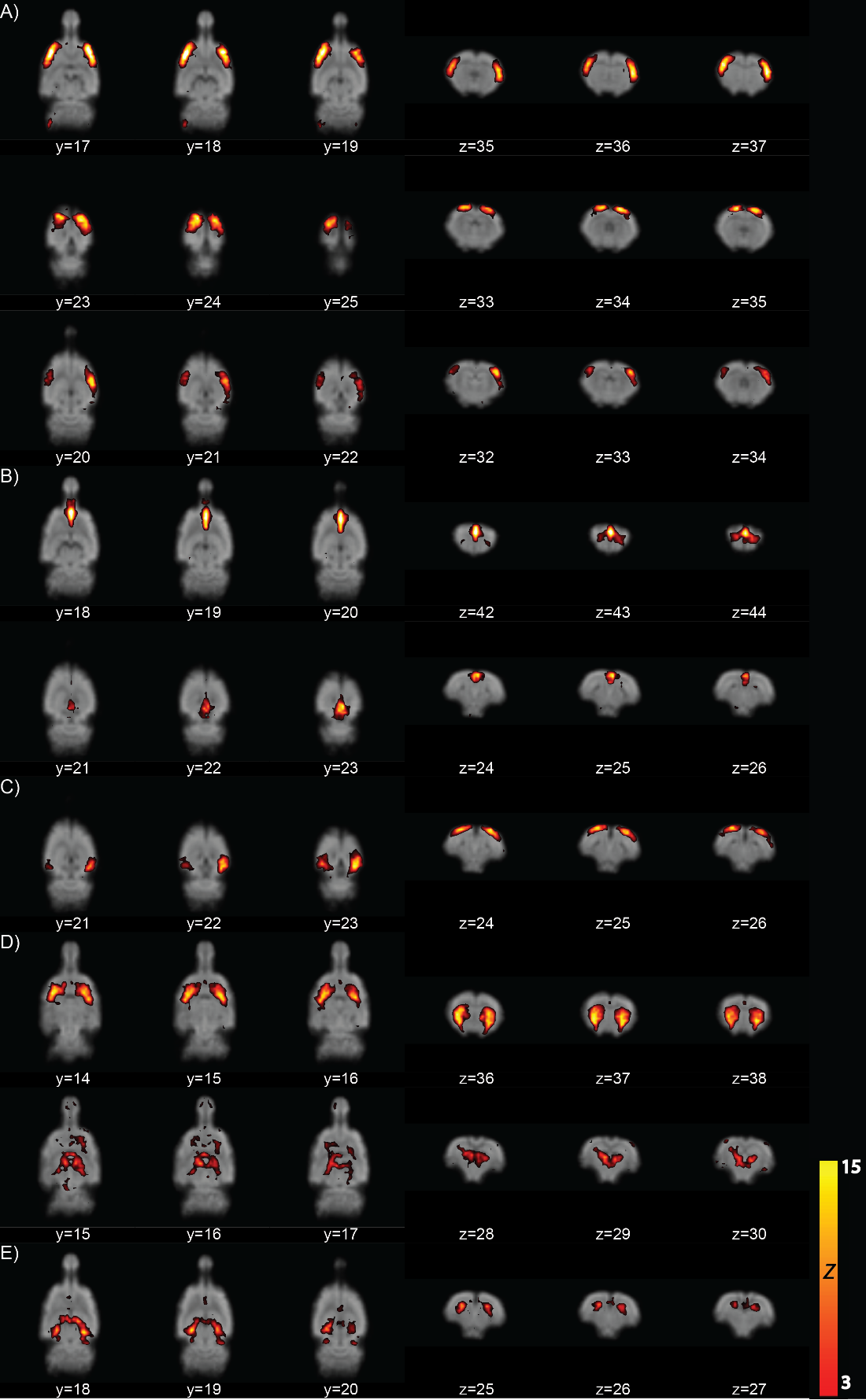


**Supplementary Figure S1: Resting-state networks identified with independent component analysis.** Functional connectivity strength of regions that are part of the sensorimotor network (A), default mode network (B), visual network (C), striatal-thalamic network (D) or hippocampal network (E) are overlaid on a mean resting-state fMRI image. Color-coding reflects Z-scores. Rat brain images are shown in axial view with y-slice numbers (left side), and in coronal view with corresponding z-slice numbers (right side).


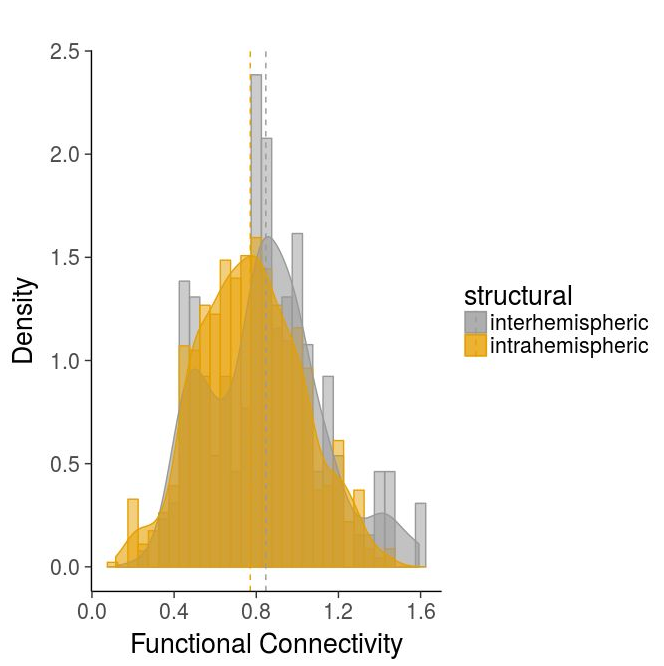


**Supplementary Figure S2: Functional connectivity density plots for interhemispheric and intrahemispheric structural connections.** Functional connectivity values are shown as Fisher’s Z-transformed full correlation coefficients. Interhemispheric connections running between both hemispheres are shown in grey, whereas intrahemispheric connections running within a hemisphere are shown in orange. The grey and orange dashed lines represent the mean functional connectivity value for the interhemispheric and intrahemispheric connections, respectively.


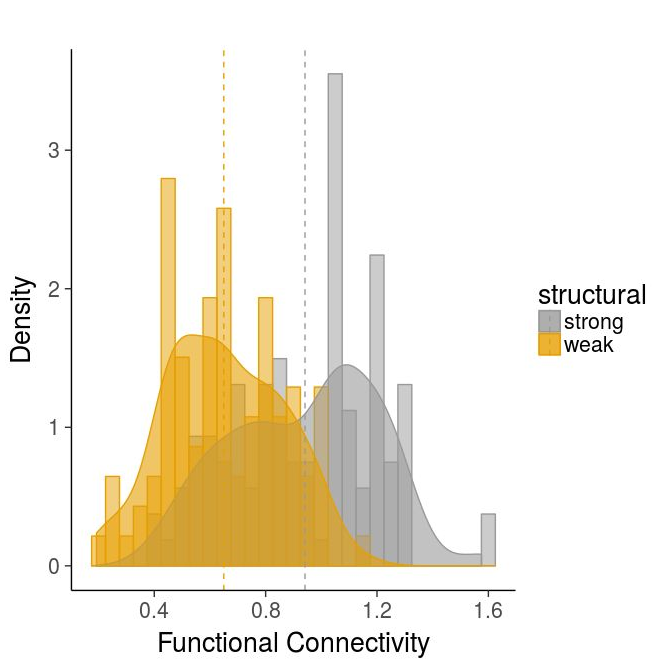


**Supplementary Figure S3: Functional connectivity density plots for strong and weak structural connections.** Functional connectivity values are shown as Fisher’s Z-transformed full correlation coefficients. The strong structural connections belong to the 25% strongest structural connections in both the macro-scale diffusion-based and the meso-scale neuronal tracer-based dataset (shown in grey). The weak structural connections belong to the 25% weakest connections at both hierarchical levels (shown in orange). The grey and orange dashed lines represent the median functional connectivity value for the strong and weak structural networks, respectively.
